# Attractive faces temporally modulate visual attention

**DOI:** 10.3389/fpsyg.2014.00620

**Published:** 2014-06-18

**Authors:** Koyo Nakamura, Hideaki Kawabata

**Affiliations:** Department of Psychology, Keio UniversityTokyo, Japan

**Keywords:** facial attractiveness, temporal attention, emotional attentional blink, emotion-induced hypervision, facial distinctiveness

## Abstract

Facial attractiveness is an important biological and social signal on social interaction. Recent research has demonstrated that an attractive face captures greater spatial attention than an unattractive face does. Little is known, however, about the temporal characteristics of visual attention for facial attractiveness. In this study, we investigated the temporal modulation of visual attention induced by facial attractiveness by using a rapid serial visual presentation. Fourteen male faces and two female faces were successively presented for 160 ms, respectively, and participants were asked to identify two female faces embedded among a series of multiple male distractor faces. Identification of a second female target (T2) was impaired when a first target (T1) was attractive compared to neutral or unattractive faces, at 320 ms stimulus onset asynchrony (SOA); identification was improved when T1 was attractive compared to unattractive faces at 640 ms SOA. These findings suggest that the spontaneous appraisal of facial attractiveness modulates temporal attention.

## INTRODUCTION

Attractive faces rapidly evoke strong affective reactions in viewers. There is considerable evidence for spontaneous appraisal of facial attractiveness; in fact, it can be assessed even when faces are presented for 13 ms (Olson and Marshuetz, 2005), or are located in peripheral vision (Guo et al., 2011). Automatic evaluations of facial attractiveness are bolstered by biological evidence, with the human brain’s reward system becoming automatically engaged – even when participants performed a task unrelated to the explicit task of judging facial attractiveness (Aharon et al., 2001; O’Doherty et al., 2003; Kim et al., 2007; Rellecke et al., 2011). This automatic appraisal of facial attractiveness impacts attentional processes, both spatially and temporally.

Attractive faces capture greater spatial attention than unattractive faces, even if appraisal of facial attractiveness is task-irrelevant. Sui and Liu (2009) reported that judgment for visual target orientation in a spatial cueing task was disrupted when an attractive face was presented in the opposite visual field. This kind of attentional capture effect was also found to affect distributed attention to multiple faces. Using a flicker paradigm – in which participants were required to report whether a face identity had changed between two alternating frames, consisting of four faces separated by a blank frame – Chen et al. (2012) showed that when all four faces were attractive, a change detection was disrupted, as opposed to unattractive faces. In addition, Liu and Chen (2012) also found that attractive faces were tracked more efficiently than unattractive faces in a multiple-object tracking paradigm. These findings indicate that spontaneous appraisal of facial attractiveness automatically modulates spatial attention, even when facial attractiveness is not related to the task at hand.

There are also several findings about the temporal characteristics of facial attractiveness perception. Research has consistently suggested that people tend to perceive attractive faces to efficiently detect potentially valuable mates. For example, people spend more time looking at attractive faces than unattractive ones (Aharon et al., 2001); additionally, the perceived duration of presentation of unattractive faces is underestimated, and less accurate, relative to attractive and neutral faces (Ogden, 2013). Recently, Arantes et al. (2013) also found that female participants overestimated the perceived duration of briefly viewed attractive male faces compared to unattractive male faces. Such biases increase the salience of an attractive face, and allow more information to be processed rapidly, leading to better detection of a potential mate. Thus, though these studies have indicated prioritized processing for attractive faces, the temporal attention modulation underlying this prioritization remains unclear.

Although no direct evidence of facial attractiveness modulating temporal attention has emerged thus far, there is much evidence on the ways in which emotional pictures or facial expressions influence temporal attention. To assess the temporal aspects of visual attention, many studies have used a rapid serial visual presentation (RSVP) procedure (Broadbent and Broadbent, 1987; Raymond et al., 1992). In this procedure, a number of visual stimuli are successively presented at identical spatial locations, typically at a rate of about 10 items/s. Participants are asked to identify two specified visual targets that are placed within the stream of distractors. Typically, attention to a second target (T2) that follows within 200–500 ms after a first target (T1) is degraded is a phenomenon known as “attentional blink (AB)” (Raymond et al., 1992; Shapiro et al., 1997). Previously, AB has been demonstrated using face image stimuli (e.g., Landau and Bentin, 2008). As seen in a variety of stimuli (usually words or pictures), attending to a T1 face impairs T2 face detection within 500 ms. Several facial attributes have been shown to influence the AB, such as facial expressions (Fox et al., 2005; Vermeulen et al., 2009; Miyazawa and Iwasaki, 2010), familiarity (Gobbini et al., 2013), gaze direction (Ricciardelli et al., 2012), and facial distinctiveness (Ryu and Chaudhuri, 2007).

A number of studies have demonstrated the effect of emotional expressions on attentional processes, in what was thought to be a form of the emotional AB (EAB; Most et al., 2005). For instance, angry faces presented as T2 tend to be detected better than happy faces (an angry superiority effect; de Jong and Martens, 2007). In some other studies, happy faces are detected better than neutral and angry faces (Miyazawa and Iwasaki, 2010). When emotional faces were presented as T1, those with fearful expressions caused a stronger AB than disgusted faces (Vermeulen et al., 2009). Likewise, happy expressions produced a stronger AB effect relative to that of fear expressions in high-anxiety participants (Fox et al., 2005). These findings suggest that both positive and negative facial expressions modulate attentional processes. Besides facial expressions, personally familiar or distinctive faces attenuate AB when these faces serve as T2 (Ganis and Patnaik, 2009; Gobbini et al., 2013).

Despite these accumulating findings, no direct evidence has thus far been provided to support the hypothesis that facial attractiveness temporally modulates visual attention. Human beings might evolve discrete mechanisms for efficiently detecting attractive faces in evolutionary processes. In our social interactions, attractive faces may be represented as a class of important social signals, which have a strong motivational influence on pervasive social behavior. If attentional mechanisms prioritize cues of an attractive face because of its importance as biological signals, attractive faces are supposed to capture greater temporal attention as facial expressions does. The aim of this study was to investigate how spontaneous appraisal of facial attractiveness modulates temporal attention. To assess the temporal characteristics of visual attention, we used the dual task RSVP (dtRSVP) procedure, in which participants were asked to identify both T1 and T2 faces embedded in a stream of face image stimuli (e.g., Zappalà et al., 2013). Specifically, we manipulated facial attractiveness of T1 faces and examined the effect of attractiveness on AB, as well as the subsequent attentional modulation. According to Ciesielski et al. (2010), an emotionally salient stimulus disturbs a neutral T2 identification from 200 to 600 ms stimulus onset asynchrony (SOA), but facilitates T2 identification performance at 800 ms. This type of late enhancement effect is referred to as emotion-induced hypervision (Bocanegra and Zeelenberg, 2009). Given that attractive faces capture greater attentional resources, we expected that the presentation of an attractive face would hinder a subsequent target (T2) identification compared to a neutral or an unattractive face, when T2 follows within 500 ms. In addition, we predicted that T2 identification performance would be temporally improved following the presentation of an attractive face at a relatively late time point, as reported in previous studies (Bocanegra and Zeelenberg, 2009; Ciesielski et al., 2010). To ascertain that these attentional modulations should only be induced by facial attractiveness, we measured subjective facial distinctiveness which often negatively correlates with facial attractiveness (Langlois et al., 1994). Facial distinctiveness has been used as the index of prototypicality of the face, described in terms of deviation from the population average of all faces (Vokey and Read, 1992). Indeed, distinctive faces are shown to be better detected among distractors during RSVP (Ryu and Chaudhuri, 2007) and remembered better than typical faces (Shepherd et al., 1991). Considering that distinctive faces are associated with greater processing efficiency, it might be that facial distinctiveness affects the magnitude of AB and hypervision. Hence, we assessed both attractiveness and distinctiveness to test the unique effect of attractiveness on attentional processes.

## MATERIALS AND METHODS

### PARTICIPANTS

Thirty-four adults (23 females; mean age, 21.2 ± 1.36 years) participated in the experiment. All participants had normal or corrected-to-normal vision and were naïve to the purpose of the study. They were individually tested in a soundproof room and paid 1000 Japanese yen for their participation. The study was approved by the local ethical committee of the Keio University, Japan. Before beginning the experiment, each participant provided informed consent and signed a written consent form.

### APPARATUS AND STIMULI

The visual stimuli were presented on a 21-inch monitor (Trinitron CPD-G420, SONY) with a refresh rate of 100 Hz, and a screen resolution of 1280 × 960 pixels; stimuli were controlled by the MATLAB program (The Math Works, Natick, MA, USA) using a MacBook Pro (MacBook Pro, Apple). Participants sat at a viewing distance of 57 cm from the monitor. Head movement was restrained using a chin rest. In this experiment, 120 white British faces (60 females) were selected from the Glasgow Unfamiliar Face Database (http://homepages.abdn.ac.uk/m.burton/pages/gfmt/; Burton et al., 2010). All faces were viewed front-on, and were emotionally neutral. Face images were adjusted to be of approximately equal size (10.5° × 13.5° in visual angle). Images were converted to grayscale and normalized to have the same mean luminance and contrast; they were presented in the center of the screen against a gray background. In our preliminary experiment, a separate group of 29 participants (21 females) rated facial attractiveness of these 120 faces on a scale of one to six (1 = least attractive, 6 = most attractive). The mean attractiveness rating score was 2.64 ± 0.68 for male faces and 2.84 ± 0.75 for female faces.

### PROCEDURE

The present experiment was divided into three parts: (i) an attractiveness rating task, (ii) a distinctiveness rating task, and (iii) a dtRSVP task.

**FIGURE 1:**
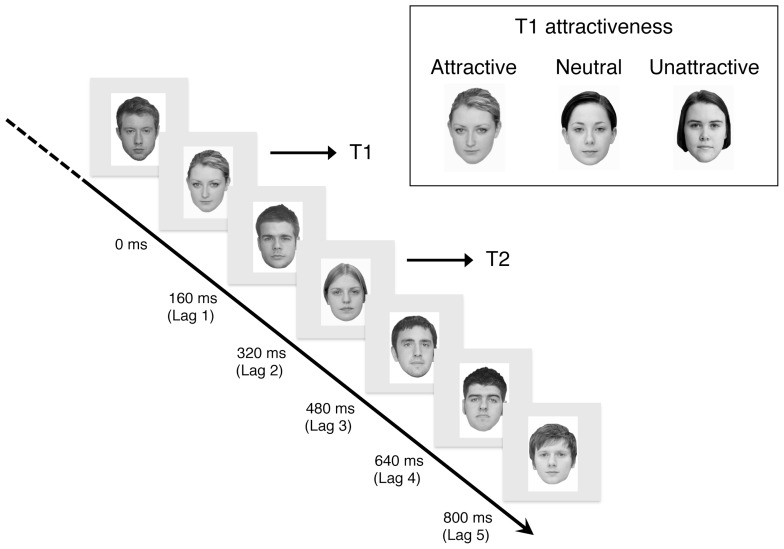
**Schematic diagram of dtRSVP task**. Note that T1 were attractive, neutral, or unattractive faces, while T2 were always neutral at 320, 640, and 800 ms time lags.

(i) In the attractiveness rating task, participants were required to rate the attractiveness of 120 faces (60 female faces) on a visual analog scale (VAS) ranging from 0 (least attractive) to 1 (most attractive). In each trial, participants clicked a mouse button to initiate the presentation of a fixation cross (500 ms), followed by the face image. Participants were able to view the face until their response was made. They were required to keep in mind that the ratings were to be made by subjective but relative judgments, and that they should be able to use the entire range of the scale across all the faces. Participants completed two separate sessions according to the gender of the faces, in which the faces were presented in a random order. The order of the sessions (i.e., gender of the faces) was counter-balanced across participants.
(ii) In the distinctiveness rating task, participants were asked to rate the distinctiveness of each female face on a VAS, ranging from 0 (least distinctive = *the face is extremely close to the average face*) to 1 (most distinctive = *the face deviates extremely from the average face*). Distinctiveness is often referred to as a proxy for prototypicality because it seems to be more meaningful to participants (Monin, 2003).
(iii) In the dtRSVP task, participants were required to identify two female targets in a stream of face images presented in rapid succession (Broadbent and Broadbent, 1987). The presentation procedure is illustrated schematically in **Figure 1**. The dtRSVP stream began with a fixation cross that was presented for 500 ms in the center of the display. The fixation cross was followed by a rapid serial presentation of 16 face images on a gray background. In each trial, there were always two female target faces (namely, T1 for the first target, and T2 for the second target) within 14 male filler faces, each presented for 160 ms. T1 was placed fifth, sixth, or seventh in the 16 face presentation. T2 was placed second, fourth, or eighth after presentation of T1; that is, T2 was presented at lags of 2, 4, or 8 faces for T1. Accordingly, SOAs between T1 and T2 were 320 ms (Lag 2), 640 ms (Lag 4), and 1280 ms (Lag 8). In the dtRSVP task, T1 stimuli consisted of three attractiveness categories: attractive, neutral, and unattractive faces. Six faces were selected for each category, in accordance with scores by each participant in the attractiveness rating task. Mean attractiveness ratings were 0.79 ± 0.13 for attractive faces, 0.42 ± 0.11 for neutral faces, and 0.15 ± 0.11 for unattractive faces. Mean rating scores for attractive faces was significantly higher than for neutral and unattractive faces (*p* < 0.001 for both), and scores for neutral faces were significantly higher than for unattractive faces (*p* < 0.001). The T2 face was randomly selected from a pool of 42 female faces that were moderately attractive (*M* = 0.43 ± 0.10), but not presented as T1. The filler stimuli were randomly selected from a pool of male faces that were rated moderately attractive (*M* = 0.42 ± 0.11). Within a trial, the same filler stimulus was not repeated. At the end of each trial, participants were asked to identify T1 and T2 from each list containing the target face, and three distractor faces. If unsure, participants were encouraged to make their best guess. Eighteen trials were repeated for each unique lag and attractiveness category combination for an overall total of 162 trials. Trials were presented in random order. To familiarize participants with the experimental task, we started with 15 practice trials using face images never used in the dtRSVP task.

## RESULTS

### ATTENTIONAL BLINK AND HYPERVISION INDUCED BY FACIAL ATTRACTIVENESS

**FIGURE 2:**
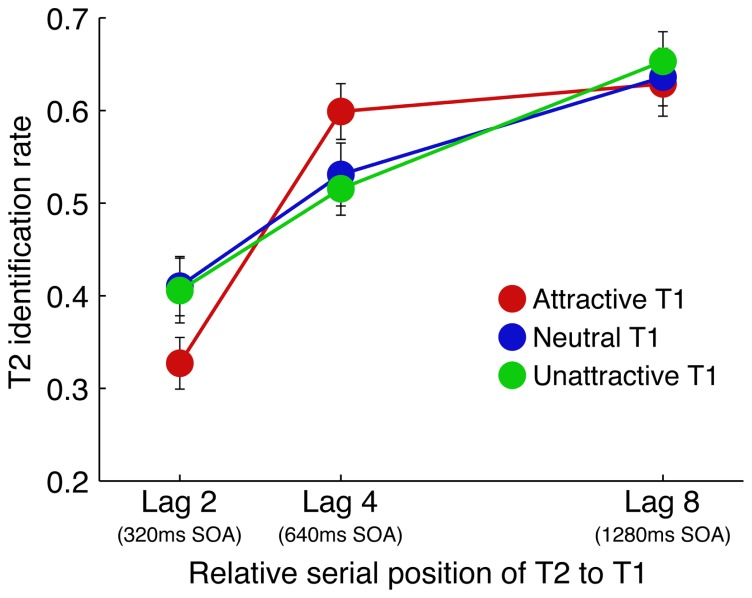
**T2 identification rates for each attractiveness category by time lag**. Error bars represent SE of the mean.

The T2 (second target) identification rate was calculated for each participant using only the trials where T1 was correctly identified. Mean T2 identification rate is shown in **Figure 2**. A 3 (T1 attractiveness; attractive, neutral, unattractive) × 3 (Lag; 2, 4, 8) ANOVA on T2 identification rate revealed a main effect of Lag to be statistically significant [*F*(2,66) = 69.65, *p* < 0.001, partial η^2 ^= 0.67], whereas that of T1 attractiveness was not [*F*(2,66) = 0.09, *p* = 0.91, partial η^2^ = 0.00]. The multiple comparisons within the Lags (Ryan’s method, *α* = 0.05) revealed that participants identified significantly fewer T2 faces at Lag 2 than at Lag 4 [*t*(66) = 7.54, *p* < 0.001] and Lag 8 [*t*(66) = 11.63, *p* < 0.001], and fewer T2 faces at Lag 4 than Lag 8 [*t*(66) = 4.09, *p* < 0.001]. More importantly, the T1 attractiveness × Lag interaction was also statistically significant [*F*(4,132) = 3.52, *p* < 0.01, partial η^2^ = 0.10]. An analysis of simple main effects revealed significant main effects of Lag at each T1 attractiveness type [attractive: *F*(2,198) = 42.49, *p* < 0.001, partial η^2^ = 0.30, neutral: *F*(2,198) = 23.64, *p* < 0.001, partial η^2^ = 0.19, unattractive: *F*(2,198) = 19.62, *p* < 0.001, partial η^2^ = 0.17] and T1 attractiveness at each Lag type [Lag 2: *F*(2,198) = 3.77, *p* < 0.05 partial η^2^ = 0.04, Lag 4: *F*(2,198) = 3.39, *p* < 0.05, partial η^2^ = 0.03]. Multiple comparisons showed participants to be significantly less accurate in identifying T2 at Lag 2 in comparison to all other Lags when T1 was attractive [Lag 2 vs. Lag 4: *t*(198) = 7.54, *p* < 0.001, Lag 2 vs. Lag 8: *t*(198) = 8.37, *p* < 0.001], neutral [Lag 2 vs. Lag 4: *t*(198) = 3.34, *p* < 0.005, Lag 2 vs. Lag 8: *t*(198) = 6.26, *p* < 0.001], or unattractive [Lag 2 vs. Lag 4: *t*(198) = 3.05, *p* < 0.005, Lag 2 vs. Lag 8: *t*(198) = 6.86, *p* < 0.001], respectively. Further, Lag 4 was significantly worse in accuracy in comparison to that of Lag 8 when T1 was neutral [Lag 4 vs. Lag 8: *t*(198) = 2.92, *p* < 0.005] and unattractive [Lag 4 vs. Lag 8: *t*(198) = 3.81, *p* < 0.001]; Lag 4 did not differ significantly from Lag 8 for accuracy when T1 was attractive [Lag 4 vs. Lag 8: *t*(198) = 0.83, *p* = 0.41]. Most importantly, further comparisons revealed that T2 identification was significantly impaired by attractive T1 faces in comparison to both neutral [*t*(198) = 2.44, *p* < 0.05] and unattractive T1 faces [*t*(198) = 2.31, *p* < 0.05] at Lag 2; T2 identification was significantly improved by attractive T1 faces compared to unattractive T1 faces [*t*(198) = 2.45, *p* < 0.01] at Lag 4. However, no effect of T1 attractiveness was observed at Lag 8 [*F*(2,198) = 0.27, *p* = 0.77].

### EFFECTS OF ATTRACTIVENESS AND DISTINCTIVENESS

To examine whether facial distinctiveness of T1 also has an effect on identifying T2, we computed a binary logistic regression equation (e.g., Monin, 2003) at each Lag for each participant, predicting whether T2 was identified or not (coded as 1 or 0) with preceding T1 attractiveness, T1 distinctiveness, and the product of these two variables capturing the interaction term. The coefficients from these binary logistic regressions were then entered as individual scores in a one-sample *t*-test. All unstandardized partial coefficients are shown in Table 1. As shown in **Table 1**, the effect of neither T1 attractiveness [*t*(32) = 1.13, *p* = 0.26, *d* = 0.19] nor distinctiveness [*t*(32) = 1.31, *p* = 0.20, *d* = 0.22] was statistically significant at Lag 2. However, the interaction coefficient was significantly lower than zero [*t*(32) = -4.02, *p* < 0.05, *d* = 0.38], suggesting that T2 identification was impaired via an interaction effect of T1 attractiveness and distinctiveness.

**Table 1:** Unstandardized partial coefficients in individual binary logistic regression equations.

**Descriptive statistics**				

	Intercept		Attractiveness		Distinctiveness		Attractiveness × Distinctiveness	
	*M*	*SD*	*M*	*SD*	*M*	*SD*	*M*	*SD*
Lag 2	-1.11	4.40	1.24	6.42	1.58	7.06	-4.02	10.66
Lag 4	-0.64	3.69	2.56	7.19	0.87	5.15	-3.16	12.94
Lag 8	0.71	3.30	-0.06	5.08	-0.04	4.68	0.58	9.96

**Inferential statistics**				

	Intercept		Attractiveness		Distinctiveness		Attractiveness × Distinctiveness	
	*t*	*p*	*t*	*p*	*t*	*p*	*t*	*p*

Lag 2	-1.47	0.15	1.13	0.26	1.31	0.20	-4.02	<0.05
Lag 4	-1.00	0.32	2.07	<0.05	0.98	0.33	-1.42	0.16
Lag 8	1.25	0.22	-0.07	0.95	-0.05	0.96	0.34	0.73

Considering partial coefficients in Lag 4, only the effect of attractiveness was significantly higher than zero [*t*(32) = 2.07, *p* < 0.05, *d* = 0.36]; the others were not significant (*p* > 0.1 for all others). This indicated that enhanced T2 identification performance following an attractive T1 at Lag 4 was due to T1 attractiveness, not T1 distinctiveness. At Lag 8, none of the partial coefficients differed significantly from zero (*p* > 0.1 for all).

## DISCUSSION

Extending the previous studies that demonstrated the spatial attentional modulation by facial attractiveness (Sui and Liu, 2009; Chen et al., 2012; Liu and Chen, 2012), we indicated that facial attractiveness temporally modulates one’s visual attention. Specifically, our results suggested that spontaneous appraisal of attractiveness of a female face target in an RSVP stream modulates temporal attention to a subsequent target face. Indeed, at early time points for T2 (i.e., Lag 2), attractive faces in T1 impaired a subsequent facial identification compared to both neutral and unattractive faces, reflecting the involuntary capture of attention by facial attractiveness. Consistent with prior research demonstrating that an emotionally salient stimulus impaired subsequent target detection (Ciesielski et al., 2010), attractive faces may capture temporal attention. Given the fact that EAB (McHugo et al., 2013) was not only found for negative valence stimuli, such as pictures inducing fear or disgust, but also for positive valence stimuli (i.e., an erotic image), it should be reasonable that facial attractiveness which may potentially have reward value captures much attention.

Furthermore, we conducted an analysis to dissociate the attentional modulation effect of facial attractiveness from that of facial distinctiveness, and confirmed that deficits in T2 identification at Lag 2 could be influenced by T1 attractiveness as a function of T1 distinctiveness. Although facial attractiveness often negatively correlated with distinctiveness (Langlois et al., 1994), these evaluations could be substantially distinguishable. For instance, prototypicality of face is not necessarily the critical determinant of facial attractiveness and most attractive faces deviate systematically from average (DeBruine et al., 2007). Thus, shifting away from a typical face (i.e., a low distinctive face) in a certain direction can increase attractiveness, while prototypicality of the face remains constant. This indicates that facial attractiveness can be differentiated from typicality or averageness. In addition, research on patients with congenital prosopagnosia (cPA), who have a specific deficit in recognizing individual faces, showed that they could evaluate facial attractiveness in spite of deficits in evaluating facial distinctiveness (Carbon et al., 2010). This finding may indicate that there are dissociable mechanisms for both facial attractiveness and distinctiveness, reflecting that these two mechanisms may diverge by the time they reach the perceptual level, and may result in the interaction between them observed in our study. It is not entirely clear why facial distinctiveness had attentional modulation effect only at Lag 2 but not any other Lags. It is possible, however, that facial distinctiveness modulates the effect of attractiveness only in a relatively early time point (i.e., Lag 2), while the effect of attractiveness endures for longer time (i.e., until Lag 4). However, whether attractiveness and distinctiveness independently impact on temporal attention remains to be seen. In our experiment, T1 faces were determined based on attractiveness but not on distinctiveness, whereas facial distinctiveness of T1 faces was not controlled. Therefore, a further study to properly control the level of attractiveness and distinctiveness is needed to examine the effect of interaction between attractiveness and distinctiveness on attentional processes.

At later a time point in T2 (i.e., Lag 4), the identification rate was improved following attractive T1 faces compared to unattractive ones. This shows the enhancement effect on T2 identification performance bestowed by attractive faces in T1. Moreover, we confirmed that facial attractiveness, but not distinctiveness, could contribute to the temporal perceptual facilitation (see Table 1). This pattern is in agreement with previous studies demonstrating emotion-induced hypervision (Bocanegra and Zeelenberg, 2009; Ciesielski et al., 2010). Ciesielski et al. (2010) reported that T2 identification performance in RSVP is moderately improved at around 800 ms SOA, when emotionally salient stimuli were presented as T1 relative to neutral stimuli. Furthermore, attractive faces might have a beneficial effect on T2 identification at a later time point, whereas it has a detrimental effect on T2 identification at the early time point. This kind of beneficial effect should be distinguished from a disengagement of temporal attention. This pattern was not observed at Lag 8 (1280 ms SOA) in this study.

The attentional modulation induced by facial attractiveness in the present study may be explained using the two-stage bottleneck model of temporal attention (Chun and Potter, 1995). This model was originally proposed to explain the temporal limit of capacities to keep visual attention to a target in an RSVP stream where the AB is produced. According to the model, temporally presented stimuli go through a form of two-stage attentional processing prior to conscious identification. Temporally presented stimuli undergo an initial stage (stage 1) that is characterized by a high capacity of perceptual and semantic processing, and a fragile visual representation. When a specific target appears, attentional resources are allocated to maintain and consolidate the stimulus representation at stage 2, in which the attentional resources are limited, and the processes are time-consuming. Therefore, when two targets are successively presented in close temporal proximity (i.e., within 500 ms), they compete for attentional resources in stage 2, frequently resulting in failure to identify T2. However, when this competition for attentional resources is diminished by increasing the temporal interval between T1 and T2, T2 identification is improved. Considering the effect of emotion on attentional process, more attentional resources may be allocated to emotional stimuli than neutral stimuli in stage 2. Thus, an emotionally salient T1 impairs subsequent T2 identification because there are fewer remaining attentional resources to process T2. In line with this explanation, our results may be interpreted as evidence that attractive T1 faces could occupy more attention than unattractive T1 faces, making few resources available for T2, and leading to impaired T2 identification. Although this interpretation fits with our data in Lag 2, the enhancement effect by attractive T1 faces at Lag 4 is not fully explained.

To explain the enhancement performance induced by an emotional stimulus in target identification, Bocanegra and Zeelenberg (2009) proposed that the emotional valence of a stimulus could trigger a general enhancement of visual processing in stage 1, and that this effect could last for a certain period of time. Thus, even after the emotional T1 face is removed, the enhancement effect carries over onto subsequent visual stimuli presented in close temporal proximity, leading to improved detection. Since this enhancement effect in stage 1 persists longer than the competition of attentional resources in stage 2, it provides improved detection of T2 in relatively later lags. Given the nature of this attentional modulation, it is possible that an increase in allocated attentional resources to an emotional T1 impairs T2 identification, while a general perceptual facilitation caused by emotional T1 improved T2 identification. Along with this interpretation, an enhanced T2 identification performance observed in this study may stem from a general perceptual facilitation in stage 1. Inferred from current data, the perceptual enhancement by facial attractiveness could last for less than 1000 ms. Taken together, our results could be interpreted in terms of the two-stage bottleneck model as follows: Attractive T1 faces capture more attention and simultaneously trigger a general perceptual facilitation in stage 1 that can last for a particular period of time. When participants attempt to identify the T1 face, more attentional resources are allocated to attractive T1 faces than neutral or unattractive ones. Thus, attentional resources for processing T2 could be restricted, leading to T2 not being identified. However, with increasing intervals between T1 and T2, visual processing for T2 in stage 1 could benefit from a general perceptual facilitation triggered by attractive T1, leading to improved identification of T2 in Lag 4. This perceptual facilitation only endures for approximately 1000 ms, and thus no attentional modulation by facial attractiveness was observed in Lag 8.

Our results provided evidence that people involuntarily evaluate attractiveness of female target faces in an RSVP stream, with temporal attention adhering to attractive faces. This finding supports the evidence that attention to emotion or attractiveness of face is rapid and automatic (Palermo and Rhodes, 2007; Sui and Liu, 2009). In the RSVP task, participants rapidly allocated greater attentional resources to an attractive face embedded in the stream of multiple faces. This is consistent with the evidence that facial attractiveness is processed even for a face presented in extremely short time (Olson and Marshuetz, 2005). Results in this experiment suggest that facial attractiveness is processed even when multiple faces are successively presented in a short time. Further, our results demonstrate that attractive faces capture temporal attention, extending previous findings regarding their attracting spatial attention. As noted in the introduction, recent research demonstrating the effect of attractiveness on spatial attention has supported that instances of such attentional bias often occur in mandatory ways (Sui and Liu, 2009; Chen et al., 2012; Liu and Chen, 2012). Indeed, these studies have shown that the appraisal of facial attractiveness is mandatory in such a way that attractive faces can compete with an ongoing task for attentional resources even in the absence of explicit judgment of facial attractiveness. This characteristic was also found in our experiment, in which attention was rapidly hijacked by attractive faces, even though the appraisal of facial attractiveness was not required explicitly in target identification. Thus, the spontaneous appraisal of facial attractiveness appears to modulate both spatial and temporal attention.

One plausible interpretation of the attentional bias to attractive faces may be provided by an evolutionary perspective. From the perspective of evolutionary psychology, the attentional bias to attractive faces might reflect an automatic reaction deeply rooted in evolution, in which people become sensitive to facial attributes constituting an attractive face due to their significance as biological signals. Attractiveness can be a sign of high genetic quality and fecundity, yielding advantages in reproduction (Johnston, 2006). Such preferences for attractive faces occur early in development, in which even infants tend to look at attractive faces for longer than unattractive ones (e.g., Langlois et al., 1991). Therefore, the perception and attentional bias of facial attractiveness may be innate (Thornhill and Gangestad, 1999). Thus, a tendency to selectively attend to attractive faces can be of advantage to an effective mating choice and peer selection. This hard-wired attentional bias to attractive faces has a profound impact on the relatively early point of cognitive processing.

In summary, our study suggests that facial attractiveness is a critical stimulus that draws much temporal attention in a short time. The findings imply that the wide range of effects of facial attractiveness on cognitive and social behavior originates from attentional bias.

## Conflict of Interest Statement

The authors declare that the research was conducted in the absence of any commercial or financial relationships that could be construed as a potential conflict of interest.
